# Comparison of Wire and Non-Wire Localisation Techniques in Breast Cancer Surgery: A Review of the Literature with Pooled Analysis

**DOI:** 10.3390/medicina59071297

**Published:** 2023-07-13

**Authors:** Shahram Shirazi, Hamed Hajiesmaeili, Muskaan Khosla, Saima Taj, Tapan Sircar, Raghavan Vidya

**Affiliations:** 1 Specialist Registrar in Breast Surgery, Princess Royal University Hospital, Kings College Hospital NHS Foundation Trust, London SE5 9RS, UK; shahram.shirazi@nhs.net; 2 Specialist Registrar in Breast Surgery, Royal Wolverhampton NHS Trust, Wolverhampton WV10 0QP, UK; 3 Senior Clinical Fellow in Breast Surgery, Royal Wolverhampton NHS Trust, Wolverhampton WV10 0QP, UK; muskaan.khosla@nhs.net (M.K.);; 4 Consultant in Oncoplastic and Reconstructive Breast Surgery, Royal Wolverhampton NHS Trust, Wolverhampton WV10 0QP, UK

**Keywords:** localisation, localization, non-palpable breast lump, breast cancer, wire-guided, wire-free, re-excision, positive margins, SAVI SCOUT, Magseed, RSL, ROLL, WGL

## Abstract

*Background and Objectives:* Wide local excision is a common procedure in the treatment of breast cancer. Wire-guided localisation (WGL) has been the gold standard for many years; however, several issues have been identified with this technique, and therefore, wire-free techniques have been developed. This scoping review synthesises the available literature comparing wire-guided localisation with the wire-free techniques used in breast-conserving cancer surgery. *Materials and Methods*: Multiple databases including Pubmed and MEDLINE were used to search articles between 1 January 2000 and 31 December 2022. Terms included “breast neoplasms”, “margins of excision”, and “reoperation”. In total, 34/256 papers were selected for review. Comparisons were made between positive margins and re-excision rates of WGL with wire-free techniques including SAVI SCOUT, Magseed, ROLL, and RSL. Pooled *p*-values were calculated using chi-square testing to determine statistical significance. *Results*: Pooled analysis demonstrated statistically significant reductions in positive margins and re-excision rates when SAVI SCOUT, RSL, and ROLL were compared with WGL. When SAVI SCOUT was compared to WGL, there were fewer re-excisions {(8.6% vs. 18.8%; *p* = 0.0001) and positive margins (10.6% vs. 15.0%; *p* = 0.0105)}, respectively. This was also the case in the ROLL and RSL groups. When compared to WGL; lower re-excision rates and positive margins were noted {(12.6% vs. 20.8%; *p* = 0.0007), (17.0% vs. 22.9%; *p* = 0.0268)} for ROLL and for RSL, respectively {(6.8% vs. 14.9%),(12.36% vs. 21.4%) (*p* = 0.0001)}. Magseed localisation demonstrated lower rates of re-excision than WGL (13.44% vs. 15.42%; *p* = 0.0534), but the results were not statistically significant. *Conclusions***:** SAVI SCOUT, Magseed, ROLL, and RSL techniques were reviewed. Pooled analysis indicates wire-free techniques, specifically SAVI SCOUT, ROLL, and RSL, provide statistically significant reductions in re-excision rates and positive margin rates compared to WGL. However, additional studies and systematic analysis are required to ascertain superiority between techniques.

## 1. Introduction

Breast cancer is now the commonest malignancy in the United Kingdom (UK), with 15% of all cancers belonging to this group, and amongst women, it accounts for 30% of all new cancer cases. Every year, there are approximately 55,500 new cases of breast cancer diagnosed in the UK according to Cancer Research UK, and surgery remains one of the key treatment options for these patients [1]. Wide local excision is a common procedure in the treatment of breast cancer, and this aims at removing a lump of breast tissue which contains the cancer and an adequate margin of healthy tissue around it. According to the Second All Breast Cancer Report, 57% of surgery for invasive and non-invasive breast cancer is now carried out in a breast-conserving manner [2]. However, for this procedure to work, it is essential to localise the cancer accurately pre-operatively.

The gold-standard technique to assist in localisation of non-palpable breast lumps has been a wire-guided one for many years. Wire-free techniques are newer and initially were based on radioactive methods; however, recently, there have been several new innovations resulting in the introduction of non-radioactive techniques in clinical practice.

**Figure 1:**
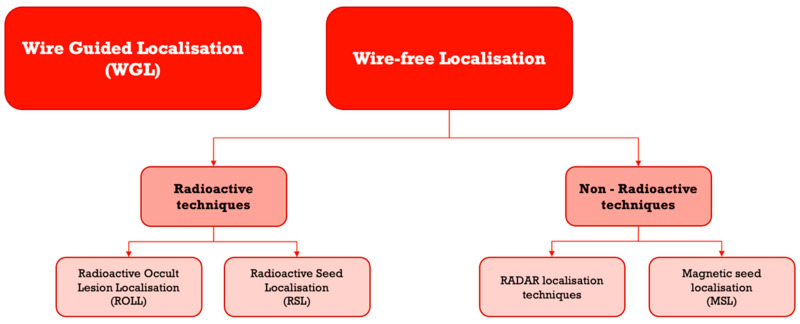
Summary of localisation techniques.

The aim of our study was to perform a scoping review of different non-wire-guided localisation techniques. We compared the main outcome of re-excision rate due to positive margin between wire-guided vs. non-wire-guided techniques in non-palpable breast cancer. Figure 1 provides a diagrammatic overview the techniques reviewed.

### 1.1. Review of Techniques

#### 1.1.1. Wire-Guided Localisation

Wire-guided localisation (WGL) was first described for accurately localising non-palpable breast lesions in 1965 by Dodd et al. [3] and was the gold-standard technique from the 1970s until recently [4,5]. The wire is typically inserted under local anaesthetic on the morning of surgery to minimise patient discomfort and potential wire migration. The radiologist visualises the lesion and guides a needle containing the wire towards the lesion, and then the wire is deployed with the hook secured within the lesion itself or in close proximity. Post-insertion imaging is performed to confirm accurate wire placement. The surgeon then uses the wire as a guide to find the lesion and excise it during wide local excision surgery.

Over the years, several issues have been identified with this technique. Wire migration can result in sub-optimal oncologic excisions and increase the need for re-excisions. The choice of wire hook shape can influence wire migration and retention within the lesion, and as such, re-excision rates of up to 52% have been reported in some case series [6,7]. Protruding wires can also interfere with the dissection route affecting margin status and re-excision rates, and wires have been shown to increase distress in patients [8]. The need for coupling of radiological and surgical services due to the requirement for same-day wire placement can also result in delays and inefficiencies in theatre utilisation [9]. Furthermore, cancellation of patients on the day of the operation and removal of these wires can be a challenge and lead to significant distress to patients. Hence, wire-free techniques for localisation have been introduced in order to tackle some of these challenges and enhance the experience for patients, radiologists, and surgeons.

#### 1.1.2. Wire-Free Localisation

Wire-free localisation techniques can be subcategorised into those that are radioactive and those that are non-radioactive. Non-radioactive techniques include RADAR, RFID, and Magnetic seed localisation and are now more commonplace in the clinical setting than radioactive techniques, which are going out of fashion in some countries.

#### 1.1.3. Radioactive Occult Lesion Localisation (ROLL)

This is a technique described in the late 1990s by Zurrida et al. [10] and Luini et al. [11]. This technique uses a radiotracer, typically Technetium-99m labelled human serum albumin (99mTc-HSA), which is injected into the lesion under ultrasound or mammographic guidance. This can be combined with a technetium injection for sentinel lymph node (SLN) identification (SNOLL) [12]. Post-injection lymphoscintigraphic imagining can be performed to confirm tracer uptake. A handheld gamma probe is used by the surgeon to detect the radiation emitted by the radiotracer, which emits an audible tone that increases in pitch and volume the closer it is to the radiotracer, providing feedback to navigate to the lesion or lymph node [13].

The advantages of ROLL over wire-guided localisation include greater comfort for patients, as there is no external wire protruding from the breast. The technique also provides greater scheduling flexibility since the radiotracer can be injected on a day prior to surgery. Identification of sentinel lymph nodes is possible too [5,13]. However, implementing ROLL in clinical practice poses challenges. There is a need for investment in gamma probes and radiation safety equipment, as well as additional training for radiology and surgical staff. The handling and disposal of radioactive materials must be performed according to institutional protocols and national regulations to ensure safety and minimise environmental impact, and this can be costly [14]. Surgeons and radiologists may also require additional training to become proficient in ROLL procedures, including using gamma probes and interpreting their signals.

#### 1.1.4. Radioactive Seed Localisation (RSL)

The RSL technique was first described in 1996 by Gray et al. [15]. An Iodine-125 or Palladium-103 titanium capsule, with a radioactive iodine seed encased within it, is used and emits low-energy gamma radiation. The seed is approximately 5 mm in size, minimising patient discomfort and tissue disruption. The seed can be injected three to four days prior to surgery, decoupling radiological and surgical services. Their half-life is 60 days, and they can be left in situ up to this time prior to removal [16]. Studies have shown that RSL seeds can successfully be implanted and detected to a depth of 6 cm [17]. A gamma probe is then used intra-operatively to localise and excise the lesion.

As with both ROLL and RSL, patients may experience anxiety regarding radiation exposure or potential allergic reactions to the radiotracer; however, studies show that the median dose rate for a single seed was low at 9.5 µSv h-1 [18]. Although rare, in RSL, there is a possibility of seed migration, requiring repositioning or additional procedures [16]. The other drawback of RSL, as with ROLL, lies with guidelines and standards associated with the management of radioactive material [19].

### 1.2. SAVI SCOUT

The SAVI SCOUT breast localisation system is more recent non-radioactive localisation technique, in use since 2014. The system consists of a 1.2 cm RADAR-based reflector, and a handheld probe that emits an electromagnetic signal to locate the reflector. The reflector is placed in the target tissue prior to surgery, under mammography or ultrasound guidance. During surgery, a probe is used to detect the reflector and provide real-time guidance to the lesion. It can be detected up to a depth of 6 cm of breast tissue [20,21,22].

The main advantage of the SAVI SCOUT system is that it is not radioactive, eliminating the challenges and costs of handling and disposing of radioactive materials. The reflector can be placed up to 30 days before surgery, thereby assisting in decoupling of radiological and surgical services [20,21]. However, implementing the SAVI SCOUT system in clinical practice can be challenging for hospitals. The system requires investment in equipment and staff training, and the scheduling and coordination of care must be adapted to accommodate reflector placement.

Intra-operative radiography of the excised tissue is performed in the operating room, and any re-excision of margins required can be carried out in the same operation if necessary. From the patient perspective, the SAVI SCOUT system offers the advantage of eliminating the need to manage an external wire, and evidence shows that the patient experience is improved compared to wire-guided techniques. Whilst there is a risk of seed migrations, this has been shown to be unlikely to happen [20].

#### 1.2.1. Magseed

Magseed technology is a wire-free, magnetic seed-based system. The seed is a small (5 mm × 0.9 mm), biocompatible, non-radioactive magnetic marker made of surgical-grade stainless steel. The seed is inserted into the lesion under ultrasound, stereotactic, or tomosynthesis mammography guidance. Post-placement imaging can detect the seed once inserted with ultrasound or mammography to confirm accurate placement. The seed can remain in situ for up to 30 days, providing flexibility in scheduling the surgery. The seeds have been shown not to migrate in previous studies. A magnetometer called a “Sentimag^®^” probe, which is connected to a console, is used by the surgeon to detect the magnetic field generated by the seed, providing real-time guidance to the lesion. The detection zone of the magnetometer is 30 mm, and the seeds can be accurately detected up to a depth of 3 cm of breast tissue [17,23,24,25].

The Magseed system offers improved comfort for patients. Patients are less anxious as there is no need for a wire protruding from their breast, and the technique is not radioactive either. The challenges associated with implementing Magseed in clinical practice include investment in equipment and staff training, and adapting scheduling and coordination of care to accommodate wire-free localisation procedures. Surgeons and radiologists may require additional training to become proficient in Magseed procedures, including using the Sentimag^®^ probe system and interpreting its signals. According to NICE, the cost of Magseed is estimated to be GBP 250 per seed (excluding the Sentimag^®^ system), which is significantly more than the cost of wire-guided localisation procedures, which are estimated to be between GBP 35 and GBP 50 [26].

#### 1.2.2. Other Techniques

RFID (radio frequency identification) tagging is another new technique that is used to localise non-palpable breast lumps. RFID tagging has been used in many industries in the past including logistics and tracking [27].

There are several manufacturers offering RFID based systems. Hologic LOCalizer is an RFID based system in use in the UK. Unfortunately, comparative data on the use of RFID based systems is currently limited, therefore RFID techniques have been kept out of scope of this review [28].

## 2. Material and Methods

We searched the Pubmed, MEDLINE, Mendeley, and Science Direct databases for articles published between 1 January 2000 and 31 December 2022 using the following search terms: “breast neoplasms” “localisation, surgical” “guidance” “radioisotopes” “ultrasound” “magnetic fields” “margins of excision” and “reoperation”.

The inclusion criteria were original research articles published in English, comparing wire-guided and wire-free localisation techniques for breast cancer surgery and reporting on one or more of the parameters of interest. The exclusion criteria were articles reporting on non-original research, articles not directly comparing wire-guided and wire-free localisation techniques, articles not reporting on the parameters of interest, and articles published before 1 January 2000. Only peer-reviewed published articles were included; these included chart reviews, retrospective and prospective cohort studies, and RCTs. Additionally, single-centre and multi-centre studies were both in the scope of this review.

The initial search identified 256 articles, of which 32 were duplicates. After screening 224 titles and abstracts, 116 articles were excluded based on the exclusion criteria. The full text of the remaining 108 articles was reviewed, and 74 articles were excluded as they did not report on the parameters of interest. Finally, 34 articles were included in this literature review, consisting of ten studies on RSL, eleven on ROLL, five on SAVI SCOUT, and eight on magnetic seed localisation.

**Figure 2:**
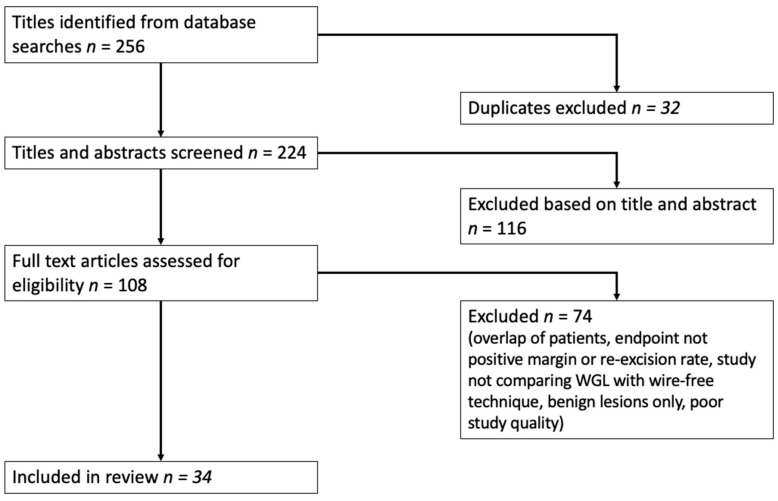
PRISMA diagram.

The PRISMA diagram in Figure 2 summarises the literature search process.

Re-excision rates and positive margins from each techniques including SAVI SCOUT, Magseed, ROLL, and RSL were compared to wire-guided techniques. Results were populated, and a pooled *p*-value was calculated using Fisher’s exact and chi-squared tests to determine their statistical significance.

### 2.1. Data Extraction

Two reviewers independently extracted data from the same selected studies for data validation purposes. A data extraction form was used which specified information that was required to be collected. This included information on publication details, patient characteristics, histology, sample sizes, localisation technique, surgical technique utilised, study design, positive margins, and re-excision rates. The data from each study were entered into a spreadsheet, which was then used for data analysis.

The main characteristics of the studies assessed have been extracted from the data analysis spreadsheet and are listed in tabulated form below in Table 1, Table 2, Table 3 and Table 4.

### 2.2. Statistical Analysis

All extracted data were tabulated and presented as percentages. Numerators and denominators were provided to address the outcomes of the included studies.

## 3. Results

In total, 34 papers directly comparing the different techniques were reviewed. Five papers were reviewed directly comparing SAVI SCOUT vs. WGL for positive margins and re-excision rates. Eleven papers were reviewed directly comparing RSL vs. WGL for positive margins and re-excision rates. Eleven papers were reviewed directly comparing ROLL vs. WGL for positive margins and re-excision rates. Eight papers were reviewed directly comparing Magseed vs. WGL for positive margins and re-excision rates.

Table 5 below summaries the number of studies included by comparator technique. The results are presented in Table 6, Table 7, Table 8, Table 9, Table 10, Table 11, Table 12, Table 13, Table 14, Table 15 and Table 16.

**Table 5:** Summary table of the included studies.

Techniques Compared	Number of Studies
SAVI SCOUT vs. WGL	5
RSL vs. WGL	11
ROLL vs. WGL	10
Magseed vs. WGL	8
Total	**34**

**Studies comparing SAVI SCOUT versus WGL.**

**Table 6:** Details of studies included in the pooled analysis for SAVI SCOUT vs. WGL.

Study	Positive Margin in SCOUT Group (*n*,%)	Re-Excision in SCOUT Group (*n*, %)	Total Number in SCOUT Group	Positive Margin in WGL (*n*, %)	Re-Excision Rate in WGL Group (*n*,%)	Total Number in WGL Group
Patel et al. [30]	4 (9.5)	3 (7.1)	42	3 (7.1)	4 (9.5)	42
Tingen et al. [31]	18 (5.6)	17 (5.3)	320	24 (13.7)	24 (13.7)	175
Choe et al. [32]	48 (18.9)	-	254	60 (17.0)	-	352
Bercovici et al. [33]	17 (8.4)	-	202	17 (13.8)	-	123
Kasem et al. [34]	-	34 (12.9)	264	-	115 (21.1)	545

**Table 7:** Comparing positive margin rate for SAVI SCOUT vs. WGL.

Study	Positive Margin in SCOUT Group (*n*,%)	Total No. in SCOUT Group	Positive Margin in WGL (*n*, %)	Total No. in WGL Group
Patel el al. [30]	4 (9.5)	42	3 (7.1)	42
Tingen et al. [31]	18 (5.6)	320	24 (13.7)	175
Choe et al. [32]	48 (18.9)	254	60 (17.0)	352
Bercovici et al. [33]	17 (8.4)	202	17 (13.8)	123
Total	87 (10.6)	818	104 (15.0)	692

**Pooled *p*-value** = 0.0105.

**Table 8:** Comparing Re-excision rate for SAVI SCOUT vs. WGL.

Study	Re-Excision in SCOUT Group (*n*, %)	Total No. in SCOUT Group	Re-Excision Rate in WGL Group (*n*,%)	Total No. in WGL Group
Patel el al. [30]	3 (7.1)	42	4 (9.5)	42
Tingen et al. [31]	17 (5.3)	320	24 (13.7)	175
Kasem et al. [33]	34 (12.9)	264	115 (21.1)	545
Total	54 (8.6)	626	143 (18.8)	762

**Pooled *p*-value** = 0.0001.

**Studies comparing RSL versus WGL.**

**Table 9:** Details of studies included in the pooled analysis for RSL vs. WGL.

Study	Positive Margin in RSL Group (*n*,%)	Re-Excision in RSL Group (*n*, %)	Total No. in RSL Group	Positive Margin in WGL (*n*, %)	Re-Excision Rate in WGL Group (*n*,%)	Total No. in WGL Group
Sharek et al. [35]	-	13 (11.4%)	114	-	15 (12.7%)	118
Gray et al. [15]	13 (25%)	-	51	26 (57%)	-	46
Lovrics et al. [16]	16 (10.5%)	23 (15.1%)	152	135 (11.8%)	29 (19.0%)	153
Tran et al. [36]	7 (2.8%)	-	254	8 (3%)	-	257
Bloomquist et al. [37]	14 (19.4%)	-	72	9 (15.3%)	-	59
Hughes et al. [38]	103 (27%)	8 (8%)	383	45 (46%)	11 (25%)	99
Langhans et al. [39]	23 (11.8%)	-	207	26 (13.3%)	-	206
Milligan et al. [40]	13 (13%)	-	100	15 (15%)	-	100
Murphy et al. [12]	33 (7.7%)	-	431	14 (5.5%)	-	256
Pieri et al. [41]	20 (8.6%)	-	233	18 (18%)	-	100
Stelle et al. [42]	16 (17%)	-	205	10 (17%)	-	155

**Table 10:** Pooled analysis for positive margin for RSL vs. WGL.

Study	Positive Margin in RSL Group (*n*,%)	Total No. in RSL Group	Positive Margin in WGL (*n*, %)	Total No. in WGL Group
Gray et al. [15]	13 (25%)	51	26 (57%)	46
Lovrics et al. [16]	16 (10.5%)	152	135 (11.8%)	153
Tran et al. [36]	7 (2.8%)	254	8 (3%)	257
Bloomquist et al. [37]	14 (19.4%)	72	9 (15.3%)	59
Hughes et al. [38]	103 (27%)	383	45 (46%)	99
Langhans et al. [39]	23 (11.8%)	207	26 (13.3%)	206
Milligan et al. [40]	13 (13%)	100	15 (15%)	100
Murphy et al. [12]	33 (7.7%)	431	14 (5.5%)	256
Pieri et al. [41]	20 (8.6%)	233	18 (18%)	100
Stelle et al. [42]	16 (17%)	205	10 (17%)	155
Total	258(12.36)	2088	306(21.4)	1431

**Pooled *p*-value** = 0.0001.

**Table 11:** Pooled analysis for re-excision rates for RSL vs. WGL.

Study	Re-Excision in RSL Group (*n*, %)	Total No. in RSL Group	Re-Excision Rate in WGL Group (*n*,%)	Total No. in WGL Group
Sharek et al. [35]	13 (11.4%)	114	15 (12.7%)	118
Lovrics et al. [16]	23 (15.1%)	152	29 (19.0%)	153
Hughes et al. [38]	8 (8%)	383	11 (25%)	99
Total	44 (6.8)	649	55 (14.9)	370

**Pooled *p*-value** = 0.0001.

**Studies comparing ROLL versus WGL.**

**Table 12:** Details of studies included in the pooled analysis for ROLL vs. WGL.

Study	Positive Margin in ROLL Group (*n*,%)	Re-Excision in ROLL Group (*n*, %)	Total No. in ROLL Group	Positive Margin in WGL (*n*, %)	Re-Excision Rate in WGL Group (*n*,%)	Total No. in WGL Group
Duarte et al. [43]	38 (59.4)	-	64	39 (60.0)	-	65
Postma et al. [44]	22 (13.6)	19 (12)	162	18 (11.8)	15 (10)	152
Thind et al. [45]	-	11 (16.0)	70	-	28 (40)	70
Ronka et al. [46]	-	7 (11.0)	64	-	3 (21)	14
Moreno et al. [47]	4 (6.6)	-	61	8 (13.6)	-	59
Medina-Franco et al. [48]	6 (12)	6 (12)	50	19 (38)	19 (38)	50
Preuss et al. [49]	-	3 (4.5)	66	-	8 (14)	57
Ocal et al. [50]	1 (1.8)	1 (1.8)	56	6 (11.5)	6 (11.5)	52
Martínez et al. [51]	7 (10.6)	-	66	12 (17.6)	-	68
Rampaul et al. [52]	-	18 (39.1)	46	-	13 (27.7)	47

**Table 13:** Pooled analysis for positive margin for ROLL vs. WGL.

Study	Positive Margin in ROLL Group (*n*,%)	Total No. in ROLL Group	Positive margin in WGL (*n*, %)	Total No. in WGL Group
Duarte et al. [43]	38 (59.4)	64	39 (60.0)	65
Postma et al. [44]	22 (13.6)	162	18 (11.8)	152
Moreno et al. [47]	4 (6.6)	61	8 (13.6)	59
Medina-Franco et al. [48]	6 (12)	50	19 (38)	50
Ocal et al. [50]	1 (1.8)	56	6 (11.5)	52
Martínez et al. [51]	7 (10.6)	66	12 (17.6)	68
Total	78 (17.0)	459	102 (22.9)	446

**Pooled *p*-value** = 0.0268.

**Table 14:** Pooled analysis for re-excision rates for ROLL vs. WGL.

Study	Re-Excision in ROLL Group (*n*, %)	Total No. in ROLL Group	Re-Excision Rate in WGL Group (*n*,%)	Total No. in WGL Group
Postma et al. [44]	19 (12.0)	162	15 (10.0)	152
Thind et al. [45]	11 (16.0)	70	28 (40.0)	70
Ronka et al. [46]	7 (11.0)	64	3 (21.0)	14
Medina-Franco et al. [48]	6 (12.0)	50	19 (38.0)	50
Preuss et al. [49]	3 (4.5)	66	8 (14.0)	57
Ocal et al. [50]	1 (1.8)	56	6 (11.5)	52
Rampaul et al. [52]	18 (39.1)	46	13 (27.7)	47
Total	65 (12.6)	514	92 (20.8)	442

**Pooled *p*-value** = 0.0007.

**Studies comparing Magseed versus WGL.**

**Table 15:** Details of studies included in the pooled analysis for Magseed vs. WGL.

Study	Positive Margin in Magseed Group (*n*,%)	Re-Excision in Magseed Group (*n*, %)	Total No. in Magseed Group	Positive Margin in WGL (*n*, %)	Re-Excision Rate in WGL Group (*n*,%)	Total No. in WGL Group
Ross et al. [53]	-	11.6% (280)	240	-	17.0% (78)	114
Kelly et al. [54]	-	14.4% (IDC)	601	-	17.7% (IDC)	608
Powell et al. [55]	-	15% (30)	200	-	21% (42)	200
Micha et al. [23]	24% (31)	17% (22)	128	20% (34)	16% (26)	168
Lenton et al. [56]	-	14.5% (9)	63	-	15.4% (8)	52
Dave et al. [57]	-	12%	946	-	13%	1170
Zacharioudakis et al. [58]	-	16% (16)	100	-	14% (14)	100
Kuhn et al. [59]	-	14.3% (2)	14	-	28.6% (4)	14
Total	24% (31)	359	2530	20% (34)		2664

**Table 16:** Pooled analysis for re-excision rate for Magseed vs. WGL.

Study	Re-Excision in Magseed Group (*n*, %)	Total No. in Magseed Group	Re-Excision Rate in WGL Group (*n*,%)	Total No. in WGL Group
Ross et al. [53]	28 (11.6)	240	19 (17.0)	114
Kelly et al. [54]	87 (14.4)	601	108 (17.7)	608
Powell et al. [55]	30 (15)	200	42 (21)	200
Micha et al. [23]	22 (17)	128	27 (16)	168
Lenton et al. [56]	9 (14.5)	63	8 (15.4)	52
Dave et al. [57]	114 (12)	946	152 (13)	1170
Zacharioudakis et al. [58]	16 (16)	100	14 (14)	100
Kuhn et al. [59]	2 (14.3)	14	4 (28.6)	14
Total	308 (13.44)	2292	374 (15.42)	2426

**Pooled *p*-value** = 0.0534.

### Summary of Results

Pooled analysis demonstrated statistically significant results for lower re-excision rates and positive margins when SAVI SCOUT was compared to WGL {(8.6% vs. 18.8%; *p* = 0.0001) (10.6% vs. 15.0%; *p* = 0.0105)}, respectively. ROLL was compared to WGL; lower re-excision rates and positive margins were noted, which were statistically significant {(12.6% vs. 20.8%; *p* = 0.0007) (17.0% vs. 22.9%; *p* = 0.0268)}, respectively. There were fewer positive margins (12.36% vs. 21.4%) and fewer re-excision rates (6.8% vs. 14.9%) in RSL compared with WGL, and the results were both statistically significant (*p* = 0.0001). The results for Magseed localisation demonstrated lower rates of re-excision than WGL; however, this was not statistically significant (13.44% vs. 15.42%; *p* = 0.0534). Only one study was identified that directly compared positive margin rates in Magseed vs. WGL. This was a small study of 296 patients in total, and it demonstrated higher rates for Magseed patients (24% vs. 20%).

## 4. Discussion

This scoping review synthesises the available literature comparing wire-guided localisation with the wire-free techniques used in breast-conserving cancer surgery. The wire-free techniques reviewed were SAVI SCOUT, ROLL, RSL, and Magseed. The pooled analysis indicates that wire-free techniques, specifically SAVI SCOUT, ROLL, and RSL, provide statistically significant reductions in re-excision rates and positive margin rates compared to WGL. These results are in line with the existing known benefits of wire-free techniques to address the known issues with WGL, such as wire displacement, decoupling of radiology and surgical services resulting in theatre inefficiencies, and reduced patient distress.

In regards to the studies comparing RSL with WGL, sample sizes ranged from small (97) to larger studies (687 patients). Out of the eleven studies analysed, several (four) were randomised control trials of high quality, two of which are multi-centric studies (level I and II evidence). Several studies do not provide data on re-excision rates, again limiting the comparability of the results. Whilst RSL has been shown to have lower positive margin rates when all ten studies reporting on this outcome were analysed, two of the studies did show higher rates in RSL, and one of these studies was a randomised controlled trial. However, the pooled analysis shows statistically significant differences in positive margin rate (*p* < 0.0001). Of the three studies reporting on re-excision rates, all demonstrated lower rates in the RSL group. These studies assessed at total of 1019 patients across both groups, and pooled rates demonstrated that RSL rates of re-excision were 8.1% less than WGL patients.

In the ROLL vs. WGL group, the quality of studies reviewed was high. Seven out of ten studies were randomised control trials, two of which were multi-centric (quality of evidence level I, II, and III). Sample sizes varied from small (78) to larger (314). Again, several studies do not provide data on re-excision rates, limiting the comparability of the results. Six out of ten studies reported on positive margins, and five of these were in favour of reduced rates in the ROLL group. In total, 905 patients across both groups were included in the analysis, and the pooled results demonstrated that positive margins were 5.9% less in the ROLL group when all studies reporting on these results were combined. Seven out of ten studies reported on re-excision rates, and five of these were in favour of the ROLL group having reduced re-excision rates. In total, 956 patients across both groups were included in the analysis, and the pooled results demonstrated that re-excision rates were 8.2% less in the ROLL group when all studies reporting on these results were combined. Despite the limitations on comparability of results, the pooled analysis shows statistically significant differences in positive margin rate (*p* = 0.0268) and re-excision rate (*p* = 0.0007) between the ROLL and WGL groups.

It is also important to note that whilst radioactive methods for localisation are going out of favour in countries such as the UK due to challenges and limitations relating to handling, disposal, and risks associated with radioactive substances, radiation safety policies vary from one country to another, and in some centres, iodine seeds still provide the gold standard of care. However, considering there are newer wire- and radioactive-free techniques for localisation, it is understandable that these techniques are starting to be preferred over radioactive methods in some places.

The studies comparing SAVI SCOUT vs. WGL included in our comparison had various sample sizes, ranging from small (84) to larger studies (842 patients). A limitation of these studies is that all of them were based on a single centre and were observational in nature, and as such, levels of evidence from these studies are classified as level III only. Kasem et al. did not include data on positive margins, and Choe et al. did not include data on re-excision rates, limiting the comparability of results. Furthermore, whilst the results for positive margins were not unanimous, with two of the studies demonstrating better positive margin rates for SAVI and the other two for WGL, the results were unanimously in favour of SAVI for re-excision rates, and pooled analysis shows statistically significant differences in positive margin rate (*p* = 0.0105) and re-excision rate (*p* = 0.0001) between the SAVI SCOUT and WGL groups. In particular, taking into account an analysis of the papers comparing re-excision rates, there was a 10.2% reduction in re-excisions in the SAVI group compared to WGL based on a total of 1388 patients from the studies.

The studies directly comparing Magseed with WGL were all observational in nature. Most were single-centre studies; however, there were three multi-centric studies, and the level of evidence was III and IV. There was one large study with a sample size of 2300 patients (Dave et al.). One study had only 28 patients across both groups. Only one study was identified that directly compared the positive margin in Magseed vs. WGL, and therefore, this limited the analysis to only re-excision rates. The one study that did report on positive margins was based on a small sample size of 296 patients in total, and it demonstrated higher rates for Magseed patients (24% vs. 20%). Further, interestingly, the Magseed technique did not demonstrate a statistically significant improvement over WGL in terms of re-excision rates, and the limited evidence available on positive margin rates warrants further research in the future on this outcome measure for this group of patients.

While our review focused on re-excision rates and positive margin rates, other factors should be considered when choosing a localisation technique. Patient satisfaction, long-term oncologic outcomes, procedural complexity, and cost-effectiveness are all relevant aspects of care that can influence the choice of a suitable localisation method.

It is important to acknowledge the limitations of the current evidence base reviewed within this scoping paper. Whilst there were several randomised controlled trials included, many studies were observational in nature, which may introduce confounding factors and limit the strength of the conclusions reached. Additionally, the literature search was restricted to articles published in English, which may exclude relevant findings published in other languages. Furthermore, the review specifically looked at positive margins and re-excision rates, and did not comprehensively assess the long-term oncologic outcomes, patient satisfaction, or cost-effectiveness of the various localisation techniques, representing an area for future research. A comprehensive systematic review or meta-analysis looking at the area of interest will provide more clarity on these areas.

## 5. Conclusions

This scoping review provides valuable insights into the clinical outcomes of various breast localisation techniques, including wire-guided localisation (WGL) and wire-free approaches of SAVI SCOUT, ROLL, RSL, and Magseed. The evidence demonstrates that wire-free techniques, particularly SAVI SCOUT, ROLL, and RSL, have advantages over WGL in terms of reduced positive margin rates and re-excision rates. However, it is important to note that not all wire-free techniques demonstrated statistically significant improvements over WGL. While Magseed localisation showed a trend toward lower re-excision rates, the difference was not statistically significant, and additional research is needed to determine the clinical implications of Magseed in terms of positive margin rates.

Over the last decade, non-wireless techniques are gradually replacing the gold-standard wire localisation. Advances in new wireless techniques are emerging for localisation of non-palpable breast cancers, and it is important to monitor their outcomes using large-scale prospective studies.

## Figures and Tables

**Table 1:** Main characteristics of the included studies (SAVI vs. WGL).

Author	Year	Comparator	Study Type	Single- or Multi-Centric	Cohort Size	Indication	Operation Performed	Histology	Level of Evidence [29]
Patel et al. [30]	2017	SAVI vs. WGL	Observational retrospective study	Single-centre	84 patients (42 SAVI, 42 WGL)	NPL (non-palpable lump)	Not specified by authors	Malignant only (on post operative histology)	III
Tingen et al. [31]	2020	SAVI vs. WGL	Observational, retrospective comparative study	Single-centre	512 (320 SAVI, 175 WGL)	NPL	Lumpectomy	Malignant and benign (on post operative histology)	III
Choe et al. [32]	2021	SAVI vs. WGL	Observational, retrospective cohort study	Single-centre	606 patients (352 WGL, 254 SCOUT)	NPL	“Breast-conserving therapy” (lumpectomy)	Malignant lesions only	III
Bercovici et al. [33]	2021	SAVI vs. WGL	Observational, retrospective study	Single-centre	525 patients (202 SAVI; 123 WGL)	NPL	Breast-conserving surgery	Malignant lesions only	III
Kasem et al. [34]	2020	SAVI vs. WGL	Systematic review and pooled analysis	Multi-centre	842 reflectors; 11 studies and authors own institutional data	NPL	Breast-conserving surgery	Malignant lesions only	II

**Table 2:** Main characteristics of the included studies (RSL vs. WGL).

Author	Year	Comparator	Study Type	Single- or Multi-Centric	Cohort Size	Indication	Operation Performed	Histology	Level of Evidence [29]
Sharek et al. [35]	2015	RSL vs. WGL	Observational, retrospective cohort study	Single-centre	232 patients (114 RSL and 118 WGL)	NPL	Lumpectomy	Malignant lesions only	III
Gray et al. [15]	2001	RSL vs. WGL	Randomised, prospective trial	Single-centre	97 patients (51 RSL, 46 WGL)	NPL	Lumpectomy or excisional biopsy	Benign and malignant	II
Lovrics et al. [16]	2011	RSL vs. WGL	Randomised, prospective trial	Multi-centre	305 patients (152 RSL, 153 WGL)	NPL	Breast-conserving surgery (lumpectomy or partial mastectomy)	Malignant (confirmed pre-operatively)	I
Tran et al. [36]	2017	RSL vs. WGL	Observational, retrospective study	Single-centre	491 patients (247 RSL, 244 WGL)	NPL	Breast-conserving surgery	Benign and malignant	III
Bloomquist et al. [37]	2016	RSL vs. WGL	Randomised, prospective trial	Single-centre	125 patients (70 RSL, 55 WGL)	NPL	Breast-conserving surgery (mastectomy excluded)	Malignant (confirmed pre-operatively)	II
Hughes et al. [38]	2008	RSL vs. WGL	Prospective trial, sequential sampling	Multi-centre	482 patients (383 RSL, 99 WGL)	NPL	Breast-conserving surgery (lumpectomy)	Benign and malignant	III
Langhans et al. [39]	2017	RSL vs. WGL	Randomised, open-label clinical trial	Multi-centre	409 patients (207 RSL, 206 WGL)	NPL	Breast-conserving surgery	Malignant (invasive breast cancer or DCIS on preoperative sonography)	I
Milligan et al. [40]	2017	RSL vs. WGL	Observational, retrospective, cohort study	Single-centre	200 patients (100 RSL, 100 WGL)	NPL	WLE (Wide local excision)	Malignant (preoperative unilateral invasive breast cancer)	III
Murphy et al. [12]	2013	RSL vs. WGL	Prospective trial, convenience sampling	Single-centre	687 patients (431 RSL, 256 WGL)	NPL	Lumpectomy	Malignant (confirmed pre-operatively)	III
Pieri et al. [41]	2017	RSL vs. WGL	Observational, retrospective, cohort study; consecutive sampling	Single-centre	333 patients (233 RSL, 100 WGL)	NPL	Lumpectomy	Malignant (confirmed pre-operatively on image guided core-biobsy)	III
Stelle et al. [42]	2018	RSL vs. WGL	Retrospective chart review	Single-centre	360 total (205 RSL, 155 WGL)	NPL	Lumpectomy	Benign and malignant	IV

**Table 3:** Main characteristics of the included studies (ROLL vs. WGL).

Author	Year	Comparator	Study Type	Single- or Multi-Centric	Cohort Size	Indication	Operation Performed	Histology	Level of Evidence [29]
Duarte et al. [43]	2015	ROLL vs. WGL	Randomised control trial	Single-centre	129 patients; 64ROLL, 65 WGL	NPL	Lumpectomy	Malignant and benign (on post operative histology)	II
Postma et al. [44]	2012	ROLL vs. WGL	Randomised control trial	Multi-centre	314 patients (162 ROLL; 152 WGL	NPL	Breast-conserving surgery	Malignant	I
Thind et al. [45]	2004	ROLL vs. WGL	Prospective study	Single-centre	140 patients (70 ROLL; 70 WGL)	NPL	Breast-conserving surgery	Benign and malignant	III
Ronka et al. [46]	2004	ROLL vs. WGL	Prospective comparative study	Single-centre	78 patients (64 ROLL, 14 WGL)	NPL	Breast-conserving surgery	Malignant	III
Moreno et al. [47]	2008	ROLL vs. WGL	Randomised control trial	Not specified by authors	120 patients (61 ROLL, 59 WGL)	NPL	Breast-conserving surgery	Benign and malignant	II
Medina-Franco et al. [48]	2007	ROLL vs. WGL	Prospective study with sequential sampling methodology	Single-centre	100 patients (50 ROLL, 50 WGL)	NPL	Breast-conserving surgery	Benign and malignant	III
Preuss et al. [49]	2021	ROLL vs. WGL	Randomised control trial	Multi-centre	123 patients (66 ROLL, 57 WGL)	NPL	Breast-conserving surgery	Malignant	I
Ocal et al. [50]	2011	ROLL vs. WGL	Randomised control trial	Single-centre	108 patients (56 ROLL, 52 WGL)	NPL	Breast-conserving surgery	Malignant and benign	II
Martínez et al. [51]	2009	ROLL vs. WGL	Randomised control trial	Single-centre	134 patients (66 ROLL, 68 WGL)	NPL	Breast-conserving surgery	Malignant only (including DCIS)	II
Rampaul et al. [52]	2004	ROLL vs. WGL	Randomised control trial	Single-centre	95 patients (48 ROLL, 47 WGL)	NPL	Breast-conserving surgery	Malignant and benign	II

**Table 4:** Main characteristics of the included studies (Magseed vs. WGL).

Author	Year	Comparator	Study Type	Single- or Multi-Centric	Cohort Size	Indication	Operation Performed	Histology	Level of Evidence [29]
Ross et al. [53]	2022	Magseed vs. WGL	Mixed prospective and retrospective cohort analysis	Single-centre	361 patients (240 Magseed, 114 WGL, 1 other)	NPL	Excisional biopsies and lumpectomies	Malignant and benign	III
Kelly et al. [54]	2022	Magseed vs. WGL	Retrospective cohort analysis	Single-centre	1221 patients (601 Magseed, 620 WGL)	NPL	Excisional biopsies and lumpectomies	Malignant and benign	III
Powell et al. [55]	2021	Magseed vs. WGL	Prospective audit	Multi-centre	200 Magseed cases compared with WGL cases in two centres over given time period (WGL case numbers not provided by authors)	NPL	Lumpectomies	Malignant and benign	III/IV
Micha et al. [23]	2020	Magseed vs. WGL	Prospective cohort study with consecutive sampling method	Single-centre	296 patients (128 Magseed, 168 WGL)	NPL	WLE (85%) and mammoplasty	Mass or calcification on pre-operative imaging	III
Lenton et al. [56]	2021	Magseed vs. WGL	Retrospective cohort analysis	Single-centre	112 patients (62 Magseed, 50 WGL)	NPL	WLE	Not specified	III
Dave et al. [57]	2022	Magseed vs. WGL	Prospective cohort study	Multi-centre	2300 patients (Magseed 946; WGL 1170 patients).	NPL	Lumpectomy, mammoplasty, axillary surgery	Dave et al. (iBRA-net study)	III
Zacharioudakis et al. [58]	2019	Magseed vs. WGL	Prospective cohort study with consecutive sampling methods	Multi-centre	200 patients (100 Magseed, 100 WGL)	NPL	WLE	Malignant (invasive and DCIS)	III
Kuhn et al. [59]	2020	Magseed vs. WGL	Prospective, non-randomised comparative cohort study	Single-centre	28 patients (14 Magseed, 14 WGL—patient choice, not randomised)	NPL	WLE	Malignant and benign	III
